# Primary breast tuberculosis mimicking breast cancer: an original study of imaging findings and differential diagnosis challenges

**DOI:** 10.25122/jml-2024-0333

**Published:** 2024-07

**Authors:** Ana-Maria Mihai, Laura Maria Ianculescu, Dragoș Crețoiu, Nicolae Suciu

**Affiliations:** 1 Alessandrescu-Rusescu National Institute for Mother and Child Health, Bucharest, Romania; 2 Ponderas Academic Hospital, Regina Maria Private Healthcare Network, Bucharest, Romania; 3 Carol Davila University of Medicine and Pharmacy, Bucharest, Romania

**Keywords:** tuberculosis, breast, differential diagnosis

## Abstract

Breast tuberculosis is a rare extrapulmonary manifestation of *Mycobacterium tuberculosis*, representing less than 0.1% of all breast pathologies in developed countries. However, in regions with high tuberculosis prevalence, such as India and Africa, its incidence is higher. The disease poses diagnostic challenges due to its ability to mimic breast carcinoma, leading to potential misdiagnosis and unnecessary surgical interventions. This study investigates the clinical and imaging characteristics of breast tuberculosis in a large cohort, with a specific focus on a rare case in a postmenopausal woman. A retrospective observational study was conducted on 1704 women who presented for mammography at the Alessandrescu-Rusescu National Institute for Mother and Child Health between 2019 and 2021. Clinical presentation, imaging results, and histopathological findings were analyzed to identify cases of breast tuberculosis. The study includes a comparative analysis with other granulomatous diseases and malignant breast conditions to highlight key diagnostic features. Among the 1704 patients, 714 (41.9%) presented with symptoms such as pain (35.4%), palpable lumps (13.2%), nipple discharge (4.3%), and breast appearance changes (2.1%). A rare case of primary breast tuberculosis was identified in a 69-year-old postmenopausal woman, presenting with a painless, palpable mass in the upper outer quadrant. Imaging demonstrated a hypoechoic mass with fine granular content and posterior acoustic enhancement, categorized as BIRADS 4A. The biopsy confirmed the diagnosis of breast tuberculosis. This study underscores the diagnostic complexity of breast tuberculosis, particularly in its ability to mimic malignancy. Through detailed imaging and clinical analysis, we emphasize the importance of biopsy in differentiating tuberculosis from breast cancer. Given the potential for misdiagnosis, clinicians should consider breast tuberculosis in differential diagnoses, especially in regions with high tuberculosis prevalence. Further research is needed to develop specific imaging criteria for earlier and more accurate diagnosis.

## INTRODUCTION

Tuberculosis is an infectious disease caused by *Mycobacterium tuberculosis*, a bacillus capable of surviving within host macrophages [1]. The breast parenchyma typically exhibits resistance to the tuberculosis bacillus [2]. Secondary breast tuberculosis may arise through retrograde dissemination from infected axillary lymph nodes or direct extension from adjacent tissues such as the ribs, sternum, shoulder joint, costochondral cartilage, or pleura into the mammary gland tissue [3,4]. Conversely, primary breast tuberculosis results from hematogenous spread following abrasions to the breast skin or through nipple fissures. In the absence of concurrent infections elsewhere in the body it represents an exceedingly rare occurrence [5,6]. The first documented case of tuberculous mastitis was reported in 1829 by the renowned English surgeon Sir Astley Cooper, who described it as a 'scrofulous swelling of the breast’ [7].

Breast tuberculosis constitutes less than 0.1% of all breast pathologies in developed nations but exhibits higher prevalence rates, ranging from 3% to 4%, in regions with elevated tuberculosis incidence, such as India and Africa [8]. In developing countries, it accounts for 3–4.5% of breast pathologies necessitating surgical intervention [9]. The demographic most commonly affected includes multiparous and lactating women aged 20 to 40 years, with additional risk factors including trauma and conditions causing immunosuppression [10]. The condition is notably rare in males, with a male-to-female ratio estimated at 1:30 [11].

The current literature lacks comprehensive imaging-based studies that document the presentation of breast tuberculosis, particularly in postmenopausal women. With breast cancer accounting for a significant portion of breast pathologies, it is crucial to delineate cases where tuberculosis mimics malignancy, as misdiagnosis can lead to unnecessary invasive procedures. This study explored the clinical presentation and imaging findings of breast tuberculosis through the analysis of a large patient cohort, including a unique case in a postmenopausal woman.

### Clinical manifestation of breast tuberculosis

The clinical manifestation of breast tuberculosis is characterized by a generally favorable systemic condition, with notable absence of constitutional symptoms such as fever, weight loss, night sweats, and anorexia [12]. Presentation varies widely; however, a predominant feature in most cases is a painless, palpable lump exhibiting irregular margins, firm consistency, and adherence to the skin or chest wall, typically localized in the upper outer quadrant or central region of the breast.

Although multiple lesions are uncommon, the clinical appearance often mimics that of carcinoma, rendering differentiation challenging [5]. Bilateral involvement has been documented in 3–30% of cases [13]. Additional clinical manifestations may include nipple and skin retraction, localized swelling, inflammatory changes, sinus formation, and axillary lymphadenopathy, albeit breast discharge is rarely observed [13]. The coexistence of carcinoma alongside breast tuberculosis is rare, posing diagnostic and therapeutic dilemmas [3]. Axillary lymph node participation is evident in 50-75% of cases, with initial presentation occasionally resembling a pyogenic abscess [14].

Given the nonspecific nature of these findings, clinical differential diagnosis remains challenging. As an example, our case report is part (and the only breast tuberculosis case) of a retrospective observational study that collected data on 1704 women who presented for mammography between 2019 and 2021 at the Alessandrescu-Rusescu National Institute for Mother and Child Health in Bucharest, Romania. Of these, 714 patients (41.9%) had symptoms at presentation. The most common symptoms included pain (35.4%), palpable breast lumps (13.2%)—5.28% in the right breast, 6.98% in the left breast, and 0.94% in both breasts—nipple discharge (4.3%), and changes in breast appearance (2.1%).

### Classification

In 1952, McKeown and Wilkinson classified mammary tuberculosis into five distinct categories: nodular tuberculous mastitis, obliterative tuberculous mastitis, sclerosing tuberculous mastitis, disseminated tuberculous mastitis, and acute miliary tuberculous mastitis [20]. In addition to this classification, Tewari and Shukla later introduced a more practical system for modern clinical use. Their classification identifies three main types of mammary tuberculosis: nodular-caseous tuberculous mastitis, disseminated tuberculous mastitis, and tuberculous mammary abscesses [2].

### Imaging

#### Chest X-ray

A chest radiograph can reveal signs of active or resolved pulmonary tuberculosis in cases of secondary mammary tuberculosis alongside clustered calcified lymph nodes in the axilla associated with mammary tuberculosis [3].

#### Mammography

Predominant mammographic findings encompass diffuse trabecular thickening and skin retraction, often presenting as an indistinct breast mass [3]. Approximately 43.5% of breast tuberculosis cases manifest as BIRADS 4/5 lesions [15]. The dimensions of tuberculous lesions depicted on mammograms typically closely correlate with clinical observations. Nodular tuberculosis may exhibit characteristics resembling a fibroadenoma [2,3].

The diffuse form often manifests as increased breast tissue density with diffuse thickening extending to the skin, simulating inflammatory carcinoma. Such presentations frequently signify heightened virulence of the infection or compromised immune response. The rare sclerosing variant predominantly affects older women [15], presenting as a dense breast mass accompanied by nipple retraction [2,3]. Asymmetry between breasts or reduction in volume and retraction of the affected breast may ensue, often leading to atrophy, indicative of delayed diagnosis or inadequate treatment [16]. Localized skin thickening and sinus formation alongside an ill-defined breast mass should prompt consideration of breast tuberculosis in the differential diagnosis. While benign calcifications (round or coarse) are commonly observed on mammography, suspicious calcifications are infrequently encountered [3].

#### Ultrasound

Given its prevalence among young women aged 20-40, ultrasound serves as the primary radiological tool for diagnosing breast tuberculosis. It is particularly efficacious in evaluating axillary lymph nodes [2,16]. Guided ultrasound is pivotal in procedures such as fine needle aspiration (FNA), core needle biopsy, or percutaneous abscess drainage [16].

#### Computed tomography (CT)

Computed tomography (CT) imaging provides valuable insights into lesions situated deep within the retromammary region and those involving the chest wall. Abscesses appear on CT as well-defined lesions with rim-like peripheral contrast enhancement. Furthermore, CT can reveal the presence of a fistula extending to the pleura or indicate involvement of infected ribs, bony structures, or lung parenchyma. Percutaneous drainage of a deep-seated abscess can be effectively performed under CT guidance [17].

#### Magnetic resonance imaging (MRI)

MRI effectively delineates sinus formations that are not discernible on ultrasound or mammography. It is also instrumental in depicting abscess extension into extramammary regions [16]. MRI is a crucial modality for visualizing the continuity of fistulous tracts into deeper tissues.

### Biopsy

There are three main methods for tissue sampling: fine needle aspiration, the most commonly used non-invasive diagnostic technique, core needle biopsy, and open biopsy [3]. Diagnosing breast tuberculosis through pathology can be challenging. However, in cases where clinical and radiological findings strongly indicate breast tuberculosis, a needle biopsy is typically sufficient for a conclusive diagnosis [2,15]. Open biopsy is rarely necessary.

### Histopathological diagnosis

Histologically, breast tuberculosis presents as granulomatous inflammation, and its differential diagnosis includes other granulomatous diseases such as idiopathic granulomatous mastitis (GM), sarcoidosis, Wegener’s granulomatosis, giant cell arteritis, actinomycosis, and fat necrosis [3]. A definitive diagnosis of tuberculous mastitis is typically based on cytological findings of epithelioid cell granulomas, lymphohistiocytic aggregates, and Langhans giant cells, with or without caseous necrosis [12,13]. Idiopathic granulomatous mastitis, characterized by multinuclear epithelioid giant cells, leukocyte infiltration, and abscess formation, lacks caseous necrosis and primarily affects breast lobules. In contrast, breast tuberculosis exhibits a more diffuse distribution of granulomas, not restricted to lobular structures. Traumatic fat necrosis histologically presents with fragmented fat globules, while plasma cell mastitis is marked by plasma cell and giant cell accumulation within dilated ducts. The presence of sulfur granules at infection sites is a distinctive histopathological feature of actinomycosis [12].

### Treatment and follow-up

The management of breast tuberculosis lacks specific medical protocols. Similar to the therapeutic approaches for pulmonary and extrapulmonary tuberculosis, breast tuberculosis typically involves a regimen of anti-tuberculosis chemotherapy lasting between 6 to 18 months. Surgical interventions such as lumpectomy or mastectomy may be considered, particularly in cases where medical treatment fails to elicit a favorable response [18]. Ultrasound-guided external drainage represents an effective modality for addressing abscesses associated with breast tuberculosis. Ultrasound-guided external drainage is also an effective option for managing abscesses related to breast tuberculosis. Most patients respond well to medical treatment, eliminating the need for additional biopsies during follow-up [17].

## MATERIAL AND METHODS

A retrospective observational study was conducted on 1704 women who underwent mammography at the Alessandrescu-Rusescu National Institute for Mother and Child Health in Bucharest, Romania, between 2019 and 2021. Clinical data and imaging results were collected and analyzed to identify cases presenting with breast tuberculosis. Patients were assessed based on imaging findings, clinical presentation, and histopathological results. This paper presents the imaging characteristics of a unique case of primary breast tuberculosis, along with a comparative analysis of the other cases diagnosed as breast carcinoma or other granulomatous diseases.

## RESULTS

Among the 1704 women included in the study, 714 patients (41.9%) presented with symptoms. The most frequent symptoms were pain (35.4%), palpable breast lumps (13.2%), nipple discharge (4.3%), and changes in the appearance of the breast (2.1%). A rare case of primary breast tuberculosis was identified in a 69-year-old postmenopausal patient. This case involved a painless, palpable mass in the upper outer quadrant of the breast. Imaging findings demonstrated an irregular hypoechoic mass with fine granular content and posterior acoustic enhancement. The lesion was categorized as BIRADS 4A, raising suspicion of malignancy. A subsequent biopsy confirmed the diagnosis of breast tuberculosis.

In this paper, we present this unique case of breast tuberculosis in a 69-year-old patient, which is atypical given the patient’s postmenopausal status, absence of hormone replacement therapy, and lack of personal or familial predisposition. The patient presented to the breast imaging department of the National Institute for Mother and Child Health - Polizu Maternity with a palpable mass in the left axillary extension, which she first noticed approximately two months earlier.

Upon physical examination, a palpable nodule measuring approximately 2.5/2 cm was identified in the upper-outer quadrant of the left breast, extending towards the axilla. No associated changes were noted in the overlying skin, and there were no clinical signs of disease involving the nipple-areolar complex or evidence of nipple discharge. The patient denied experiencing fever, cardiovascular, or respiratory symptoms, and routine blood and urine analyses revealed results within normal limits. Furthermore, there was no reported history of pulmonary disease.

Subsequent diagnostic evaluation included bilateral mammography with tomosynthesis and bilateral ultrasound to further assess the clinical findings.

**Figure 1:**
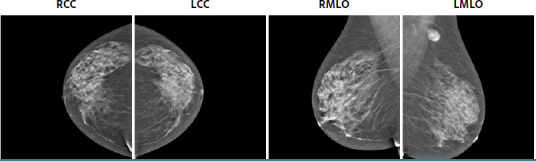
The mammographic assessment revealed an opacity extending into the left axillary region, characterized by a predominantly clear outline, partially obscured margins, and exhibiting a slight suspicion level categorized as BIRADS 4A

**Figure 2:**
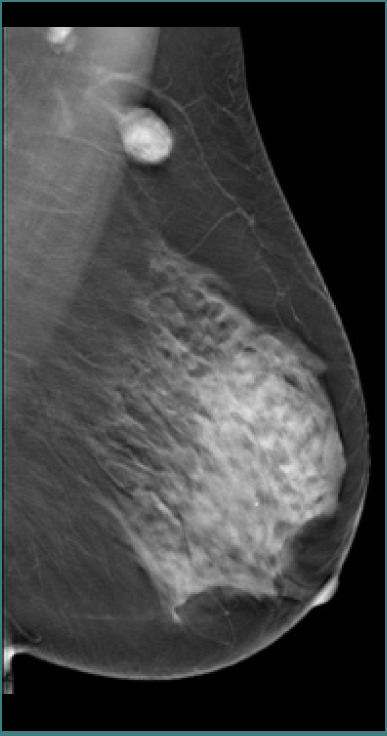
Mammographic findings with tomosynthesis show axillary adenopathy at the periphery of the imaging field

The palpable lesion identified clinically manifests as opacity in the left axillary extension upon mammographic assessment, characterized by a primarily clear contour that is partially obscured, raising slight suspicion, and categorized as BIRADS 4A (Figures 1 and 2). A significant proportion (43.5%) of breast tuberculosis cases present as BIRADS 4/5 lesions, complicating the distinction from breast carcinoma [15].

**Figure 3:**
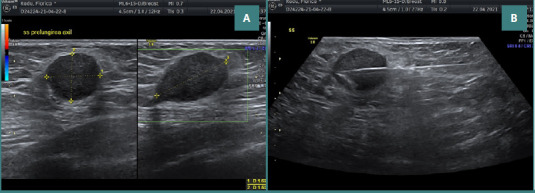
A, The ultrasound characteristics of the lesion revealed a hypoechoic image displaying fine granular content, homogeneity, and a posterior enhancement phenomenon. The contour is slightly irregular in localized areas, and no Doppler signal was detected. B, The lesion was classified as BIRADS 4A, requiring an ultrasound-guided biopsy

**Figure 4:**
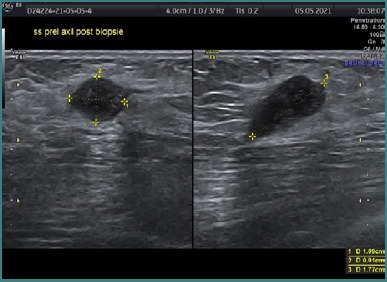
Post-biopsy ultrasound assessment shows a flattened image with observed slight extravasation of dense content upon withdrawal of the puncture needle

The ultrasound assessment depicted a hypoechoic image characterized by fine granular content and homogeneity, accompanied by a posterior acoustic enhancement. The lesion exhibited a slightly irregular contour in localized regions and lacked a Doppler signal, categorizing it as BIRADS 4A (Figure 3A). A puncture biopsy procedure was conducted (Figure 3B). Immediately following the biopsy, there was a noticeable flattening of the image corresponding to the breast lesion, with a small extravasation of dense content evident upon withdrawal of the puncture needle (Figure 4).

The histopathological analysis of the breast puncture-biopsy fragments revealed connective adipose and vascular tissue devoid of ductal-acinar structures. The specimens had varying degrees of chronic granulomatous inflammation characterized by multiple confluent granulomas comprising Langhans-type multinucleated giant cells, lymphocytes, and epithelioid cells forming palisades around central areas of caseous necrosis.

**Figure 5:**
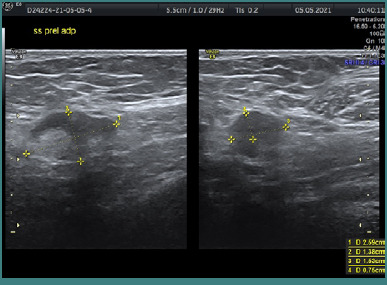
Ultrasound-guided punctured axillary adenopathy

Ipsilateral axillary adenopathy manifests in 20–69% of breast tuberculosis cases [15,16]. These lymph nodes typically exhibit a rounded or oval shape with smooth margins and enlargement (short axis > 1 cm or cortical thickness > 5 mm) [14]. Distinguishing features include a fatty, echogenic hilus and an oval shape, aiding differentiation from malignant lymphadenopathy [16]. In our patient’s case, ipsilateral adenopathy was detected and subsequently punctured under ultrasound guidance (Figure 5).

**Figure 6:**
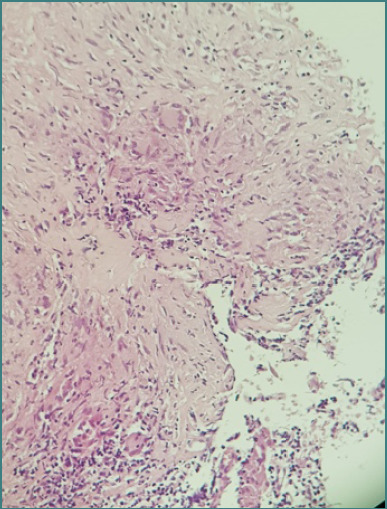
Histopathological examination

Histopathological analysis of the left axillary adenopathy revealed lymph node tissue with disrupted architecture characterized by extensive fibrosis and sclerohyaline areas interspersed with zones of organized necrosis (Figure 6). Additionally, sporadic granulomas containing multinucleated giant cells with eosinophilic cytoplasm, some exhibiting nuclei arranged in a crown or horseshoe pattern at the periphery, were identified at various levels.

## DISCUSSION

Tuberculosis represents a significant public health challenge within the European Union (EU) and the European Economic Area (EEA), notably affecting marginalized populations [19]. Romania, in particular, exhibits the highest tuberculosis incidence rates within the EU/EEA, comprising nearly a quarter (23.4%) of reported cases in 2017, with notification rates six times greater than the EU/EEA average [20,21].

The diagnostic challenge of breast tuberculosis arises due to its clinical resemblance to carcinoma, bacterial abscesses, and other granulomatous diseases, such as idiopathic granulomatous mastitis, which are also on the rise. Given the paucibacillary nature of breast tuberculosis, conventional diagnostic tests such as microscopy, culture, and nucleic acid amplification tests lack sufficient sensitivity compared to those used in pulmonary tuberculosis [22]. Consequently, breast TB often faces misdiagnosis as a nonspecific abscess or carcinoma [15].

Antituberculosis therapy (ATT) constitutes the cornerstone of medical management for breast tuberculosis. However, specific guidelines tailored to breast TB chemotherapy remain elusive, necessitating adherence to protocols established for pulmonary tuberculosis. ATT achieves successful outcomes in nearly 95% of cases, typically administered over a six-month regimen comprising initial treatment with isoniazid, rifampicin, pyrazinamide, and ethambutol, followed by isoniazid and rifampicin for the subsequent four months. Surgical interventions such as drainage of breast abscesses, biopsy of abscess walls, sinus scraping, or incisional/excisional biopsies are minimally invasive options. Small lesions often respond well to excisional biopsy complemented by full-course ATT. Surgical excision may be required for residual nodules post-ATT. In rare instances of extensive disease involving large, ulcerated masses and draining axillary lymph nodes, necessitating organ preservation, radical interventions such as simple mastectomy with or without axillary clearance may be considered. Concurrent management of breast cancer dictates surgical approaches contingent upon the stage of malignancy [23].

## CONCLUSION

In conclusion, this study adds to the growing body of evidence suggesting that breast tuberculosis can present similarly to breast carcinoma, complicating diagnosis. Through the detailed analysis of a rare postmenopausal case and the broader cohort of patients, we highlight the importance of imaging and biopsy in distinguishing between the two. Clinicians should maintain a high index of suspicion for breast tuberculosis in appropriate clinical settings, particularly in endemic regions. Further research is necessary to develop more specific imaging criteria that could aid in the earlier differentiation of breast tuberculosis from malignancies.
